# Supplementary material for: Global hypo-methylation in a proportion of glioblastoma enriched for an astrocytic signature is associated with increased invasion and altered immune landscape
Source: eLife. 2022 Nov 22;11:e77335. doi: 10.7554/eLife.77335 (PMC9681209; doi:10.7554/eLife.77335)
Supplement: Figure 2—source data 1. [file elife-77335-fig2-data1.zip › Figure_2_source_data_1/Figure_2C/homerResults/motif22.similar.html]

motif22

## Information for motif22

C
G
A
T
A
T
C
G
A
G
T
C
A
G
T
C
C
G
T
A
A
G
T
C
G
C
A
T
A
C
T
G
G
T
C
A
A
G
T
C
A
G
T
C
A
C
G
T
  
Reverse Opposite:  

C
G
T
A
A
C
T
G
A
C
T
G
A
C
G
T
A
G
T
C
C
G
T
A
C
T
A
G
A
C
G
T
C
T
A
G
A
C
T
G
A
T
G
C
C
G
T
A
  

|  |  |
| --- | --- |
| p-value: | 1e-14 |
| log p-value: | -3.244e+01 |
| Information Content per bp: | 1.842 |
| Number of Target Sequences with motif | 13.0 |
| Percentage of Target Sequences with motif | 1.11% |
| Number of Background Sequences with motif | 2.7 |
| Percentage of Background Sequences with motif | 0.06% |
| Average Position of motif in Targets | 89.4 +/- 56.3bp |
| Average Position of motif in Background | 119.1 +/- 59.6bp |
| Strand Bias (log2 ratio + to - strand density) | 1.8 |
| Multiplicity (# of sites on avg that occur together) | 1.38 |
| Motif File: | file (matrix) reverse opposite |

### Similar de novo motifs found

|  |  |  |  |  |  |  |  |
| --- | --- | --- | --- | --- | --- | --- | --- |
| Rank | Match Score | Redundant Motif | P-value | log P-value | % of Targets | % of Background | Motif file |
| 1 | 0.752 | T G A C C G T A A C T G A G T C A G T C G T C A A G T C A C G T A C T G G T C A A G T C A G T C C G T A A G C T A T G C | 1e-12 | -29.505524 | 0.85% | 0.04% | motif file (matrix) |
| 2 | 0.605 | A C T G A G T C A T C G C G T A C T A G T G A C G A T C C G A T A T G C A C G T A C T G G C T A A T G C A G T C G A C T C A G T A T C G C T G A A T C G G A T C T A C G C G T A A C T G A T G C A T G C | 1e-11 | -25.778830 | 0.77% | 0.01% | motif file (matrix) |
| 3 | 0.734 | A T C G G T A C A T C G C T G A A C T G A G T C A T G C G T C A A G C T C A G T A T C G G T C A T A G C G T A C A G C T A G T C C T A G C G T A C A T G G T A C | 1e-9 | -22.157850 | 0.68% | 0.02% | motif file (matrix) |
| 4 | 0.898 | A G T C A G T C C G T A A G C T A C G T A C T G G C T A A G T C A G T C A C G T | 1e-6 | -15.285174 | 0.51% | 0.03% | motif file (matrix) |
